# Squamous differentiation in pT1 bladder urothelial carcinoma predicts poor response for intravesical chemotherapy

**DOI:** 10.18632/oncotarget.18563

**Published:** 2017-06-19

**Authors:** Gang Li, Jieping Hu, Yuanjie Niu

**Affiliations:** ^1^ Department of Urology, The Second Hospital of Tianjin Medical University, Tianjin Institute of Urology, Tianjin, 300211, China; ^2^ Department of Urology, The First Affiliated Hospital of Nanchang University, Nanchang, Jiangxi, 330006, China

**Keywords:** squamous differentiation, pT1, bladder cancer, urothelial carcinoma, intravesical chemotherapy

## Abstract

The role of squamous differentiation in pT1 bladder tumors in the response to intravesical chemotherapy was unknown. We performed a retrospective analysis of 213 pT1 bladder urothelial carcinoma patients with squamous differentiation (group1), the remaining 213 pT1 pure urothelial carcinoma served as controls (group2). All cases were treated with transurethral resection of bladder tumor and subsequent intravesical chemotherapy. Within a five-year period, the tumor recurrence rate was 75.1% (160/213) in group 1 and 64.3% (137/213) in group 2. Tumor grade (HR = 2.926, *P =* 0.014), number of tumors (HR = 2.130, *P =* 0.038), tumor size (HR = 2.748, *P =* 0.031), and squamous differentiation (HR = 3.726, *P =* 0.019) were found to be important prognostic factors. Subgroup analysis for high grade tumors was performed, finding that group 1 had higher recurrence rate (50.3% vs 36.3%; for group 2). Progression was found in 32.2% (30/160) of group1 and 15.1% (11/137) of group2 (*P =* 0.011). Our data suggests that squamous differentiation is a predictor of poor response for intravesical chemotherapy, and that early radical cystectomy should be performed for high grade tumors, especially when dealing with recurrent cases.

## INTRODUCTION

Urothelial carcinoma of the bladder (UCB) demonstrates a wide range of clinical behavior and morphology, including a peculiar capacity for divergent histologic differentiation, in which glandular and squamous have, in particular, been widely reported in UCB [1, 2]. Focal squamous differentiation is seen most frequently with high-grade urothelial carcinoma, with some reporting this in 20–50% of urothelial carcinoma cases [3]. Keratinization and/or intercellular bridges are classical morphological features which can be found in squamous differentiation (SD) [4]. Squamous patterns detected in the histopathological specimen represents a negative prognostic factor [5, 6]. It reported that the presence of a squamous component in patients with UCB may be associated with an ominous outcome treated with radical cystectomy [7]. An intensive case-control analysis suggested, however, that the outcomes of UCB patients who underwent radical cystectomy with SD were similar to those of patients facing pure UCB [1]. In a small study, patients with squamous tumors had lower five-year survival rates in comparison to patients with conventional high-grade tumors (79.1% VS 89.5%, respectively) treated with bacillus Calmette–Guerin (BCG) [8]. Moreover, patients with SD were more likely to have extravesical tumors and node positive disease [9, 10]. These factors may alter the outcome of SD in patients. Thus, we conducted a retrospective study in order to observe tumor recurrence and progression to measure the efficacy of intravesical chemotherapy in T1 UCB with or without SD.

## RESULTS

### Patient characteristics

The main characteristics of all patients are listed in Table 1. The mean age is 67.1 ± 12.8 years old for all patients, and 67.2 ± 12.7 and 67.0 ± 12.8 for group 2 and 1. respectively. Males composed 80.5% of all patients, and males accounted for 79.8% of group 2 and, 81.2% of group 1. There was no significant difference in gender distribution (*P =* 0.716). In group 2, 128 (60.1%) patients had high grade tumors, 61 (28.6%) had multiple tumors, and 73 (34.3%) had tumors larger than 3 cm in diameter. In group 1, these categories were 149 (70.0%), 65 (30.5%), 66 (31.0%), respectively. Individually, tumor grading was statistically significant in comparison with group 2 (*P =* 0.042). The tumor recurrence rate over a five-year period was 64.3% (137 patients) in group 2 and 75.1% (160 patients) in group 1 (*P =* 0.020).

**Table 1:** The main characteristics of the patients

Characteristic		Total	PUCB	SUCB	*P* value
Cases, *n*		426	213	213	
Mean age (range), years		67.5 (28–92)	67.5 (31–90)	67.6 (28–92)	0.771
Gender, *n* (%)	M	343	170	173	0.716
	F	83	43	40	
Tumor grade, *n*	Low	149	85	64	0.042
	High	277	128	149	
Tumor multiplicity, *n*	No	300	152	148	0.750
	Yes	126	61	65	
Tumor size (cm), *n*	< 3	287	140	147	0.535
	≥ 3	139	73	66	
Recurrence, *n*	No	129	76	53	0.020
	Yes	297	137	160	
Progression, *n*	No	364	193	171	0.003
	Yes	62	20	42	

PUCB: pure urothelial carcinoma of bladder; SUCB: urothelial carcinoma of the bladder with SD.

### Outcome for single-factor analysis

Patients’ follow-up period ranged from 4 to 112 months with an average of 62.2 months, and 297 (69.7%) of all 426 patients had at least one recurrence, while 62 (20.9%) of 297 patients progressed to muscle invasive disease during follow-up period. Progression was observed in 62 (14.6%) patients, 20 of whom (9.40%) were from group 2 and 42 of whom (19.7%) were from group 1 (*P =* 0.004). A chi-square test was used to test the significant differences between single factors and recurrence. The results are shown in Table 2. Significant differences were found regarding tumor grade, size, SD, and the number of tumors in terms of recurrence.

**Table 2:** Chi square test of single factors

Variable	Non Recurrence	Recurrence	λ^2^ Value	*P* Value
Age				
≤ 67	65	149	0.002	1.000
> 67	64	148		
Gender				
Male	104	239	0.001	1.000
Female	25	58		
Grade				
Low	55	94	4.773	0.035
High	74	203		
Number				
Single	102	198	6.642	0.011
Multiple	27	99		
Size				
< 3 cm	96	191	4.181	0.043
≥ 3 cm	33	106		
Squamous				
No	76	137	5.882	0.020
Yes	53	160		

### Multiple logistic regression analysis

Factors that considered significant differences associated with recurrence according to the chi-square test were investigated using a multiple logistic regression analysis. Variables that represented tumor grade, number of tumors, and tumor size were included. Age, gender and SD were likewise not retained in the final models, as they were not significant at the 5% level and their inclusion did not improve either model’s discrimination or calibration. The following variables were identified to be of prognostic importance in the univariate analysis: grade (HR = 2.926, *P =* 0.014), number of tumors (HR = 2.130, *P =* 0.038), tumor size (HR = 2.748, *P =* 0.031), SD (HR = 3.726, *P =* 0.019).

### Subgroup analysis variables with recurrence

To explore the role of SD, we compared the recurrence rate between group 2 and group 1. As indicated in Table 3, 117 (78.5%) of 149 high tumor grade patients had recurrence in group 1, while only 86 (67.2%) of 128 high tumor grade patients had recurrence in group 2 (*P =* 0.041). Comparing group 2 with group 1 under the consideration of variables such as age and gender revealed no significant difference.

**Table 3:** Chi square test in subgroup

	PUCB		SUCB		
	NR	R	NR	R	*P* value
Age					
≤ 67	37	68	28	81	0.139
> 67	39	69	25	79	0.072
Gender					
Male	61	109	45	128	0.061
Female	15	28	8	32	0.148
Grade					
Low	34	51	21	43	0.396
High	42	86	32	117	0.041
Number					
Single	51	101	51	97	0.903
Multiple	18	43	19	46	1.000
Size					
< 3	48	92	48	99	0.803
≥ 3	19	54	14	52	0.553

NR: Non recurrence; R: recurrence; PUCB: pure urothelial carcinoma of bladder; SUCB: urothelial carcinoma of the bladder with SD.

### Progression

Sixty-two of 297 recurrent patients (20.9%) progressed to muscle invasive disease. Forty-two (67.7%) of 62 were SD patients, and 20 (32.3%) patients were pure UCB, aAs indicated in Table 4. In the high-grade tumor group, 30.8% (36 out of 117) patients with SD experienced tumor progression, whereas, only 17.4% (15 out of 86) group 2 patients had tumor progression (*P =* 0.034). In the low-grade tumor group, no significant difference was found between group 2 and group 1 (*P =* 0.749).

**Table 4:** The progression patients in subgroup

	Low		High		Total
	NP	P	NP	P	
PUCB	46	5	37	6	94
SUCB	71	15	81	36	203
Total	117	20	118	42	297

NP: Non progression; P: progression.

## DISCUSSION

Worldwide, there are approximately 430,000 new bladder cancer diagnoses annually [11]. Urothelial carcinoma (UC) accounts for the majority of bladder cancer diagnoses and has a substantial impact on public health. The morphological diversity of UC is common. UC with SD is the most common variant which exists mainly in muscle-invasive UC, and there is mounting evidence revealed that SD may represent a precursor to invasive bladder cancer [8]. We detected SD in tumors via haematoxylin-eosin (H&E) stains and immunohistochemical (IHC) and explored SD’s association with tumor progression.

Our data indicated that the important prognostic factors for recurrence are: tumor grade, number of tumors, tumor size, and SD. SD was related to tumor recurrence and was also associated with tumor progression (Table 1), especially for high grade tumors (Table 3). We noted that patients in our study were all received intravesical chemotherapy with epirubicin or hydroxycamptothecine but not BCG, mitomycin C (MMC), or doxorubicin. In particular, BCG was not available in China because it has not been permitted by the China Food and Drug Administration. Our results were consistent with other studies which found that tumor grade, number of tumors, and tumor size affected disease outcome [12]. Rianne concluded that multiple tumors increased the risk of recurrences by 1.56-1.8 in comparison with single tumors in Dutch cohort (*n =* 724) and Spanish cohort (*n =* 137) [13]. Similarly, in high-risk non-muscle-invasive bladder cancer patients treated with adjuvant BCG, tumor size was found to correlate with recurrence and progression [14]. To our best knowledge, it is the first time to prompt that SD can predict poor response to intravesical chemotherapy in pT1 bladder urothelial carcinoma.

It is well known that each histological variant has a different biology, data from 361 patients with invasive bladder carcinoma suggests that conventional urothelial carcinoma, SD carcinoma and pure squamous carcinoma presented distinct outcomes, as mean survival periods were 73, 36, and 9.4 months, respectively [5]. We demonstrated that SD also predicts a poor response to intravesical chemotherapy with epirubicin or hydroxycamptothecine outcome in pT1 bladder cancer. When comparing patients with squamous cell carcinoma and those with SD who were treated with radical cystectomy and pelvic lymph node dissection, there appears to be no evidence of a difference in cancer specific survival or overall survival (log rank overall survival *P =* 0.6, cancer specific survival *P =* 0.17) [15]. SD in UCB were more likely to represent the biological characteristic of squamous cell carcinoma.

The strategy to battle UCB includes transurethral resection of bladder tumor (TURBT), intravesical chemotherapy, and radical cystectomy. Akdas reported a case of squamous cell carcinoma of the bladder induced by radiation therapy [16], thus radiation may avoid in pT1 UCB. For patients with pure squamous cell, adenocarcinoma, or small cell carcinoma, there is clear evidence to alter treatment paradigms [17]. In the subgroup of tumor grade, our data indicated that patients who suffered from high grade tumor accompanied by SD had a higher recurrence rate (78.5% vs. 67.2%, *P =* 0.041, group 1 vs. group 2; Table 3) and progression rate (30.8% vs. 17.4%, *P =* 0.034, group 1 vs. group 2; Table 4), supporting the idea that patients with high-risk non-muscle-invasive tumors and variant histology should be offered early cystectomy [18], especially for patients harboring SD.

Limitations of this study are noted as follows: (1) nonrandomized, retrospective analysis of a single center, which decreased the level of evidence; (2) the quality of TURBT operated by distinct surgery can affect the outcome; (3) two drugs (epirubicin and hydroxycamptothecine) applied in this study, distinct effect may achieved by different drug, we did not conducted detailed analysis; (4) there were some other intravesical chemotherapies like BCG or MMC in non-muscle-invasive bladder cancer [19], but were not included in our study, Scosyrev concluded presence of squamous or glandular differentiation in locally advanced UC of the bladder does not confer resistance to methotrexate, vinblastine, doxorubicin and cisplatin (MVAC) [20], we do not ensure group 1 obtain same outcome in other chemotherapy. (5) we excluded the patients presence of CIS, which was an important prognostic factors [21].

## MATERIALS AND METHODS

### Patients

We retrospectively analyzed the clinical and pathological data of 213 patients who were diagnosed as pT1 bladder urothelial carcinoma with SD from May 2001 to Mar 2016 in our institution. Moreover, 213 pT1 urothelial carcinoma cases without SD were collected as control. The pathologic stage was based on the 2009 Union for International Cancer Control (UICC) TNM staging system. Tumor grade was based on the 2004 World Health Organization (WHO) grading system for non-invasive urothelial neoplasia [22]. Eligible cases meet the following criteria: (1) all patients should be confirmed as group 2 or UCB with SD by at least two pathologists; (2) all patients treated with TURBT; (3) patients who underwent schedule intravesical chemotherapy with epirubicin 50 mg or hydroxycamptothecine 30 mg once a week for 8 weeks and then once a month up to one year; (4) cystoscopy was given during the postoperative follow-up according to the European and US guidelines; (5) recurrence patients who received TURBT and intravesical chemotherapy in the low grade group and radical cystectomy in the high grade group. The exclusion criteria for our analysis were: (1) Ta, Tis or muscle invasive tumors; (2) imaging data indicated pelvic lymph nodes or distant organs metastases; (3) patients with a history of previous urothelial carcinoma or concomitant upper urinary tract urothelial carcinoma; (4) glandular, lymphoepithelioma-like, sarcomatoid or other differentiation; (5) Patients underwent partial or radical cystectomy; (6) patients underwent irregular intravesical chemotherapy or loss of follow up.

### Pathology

**Figure 1:**
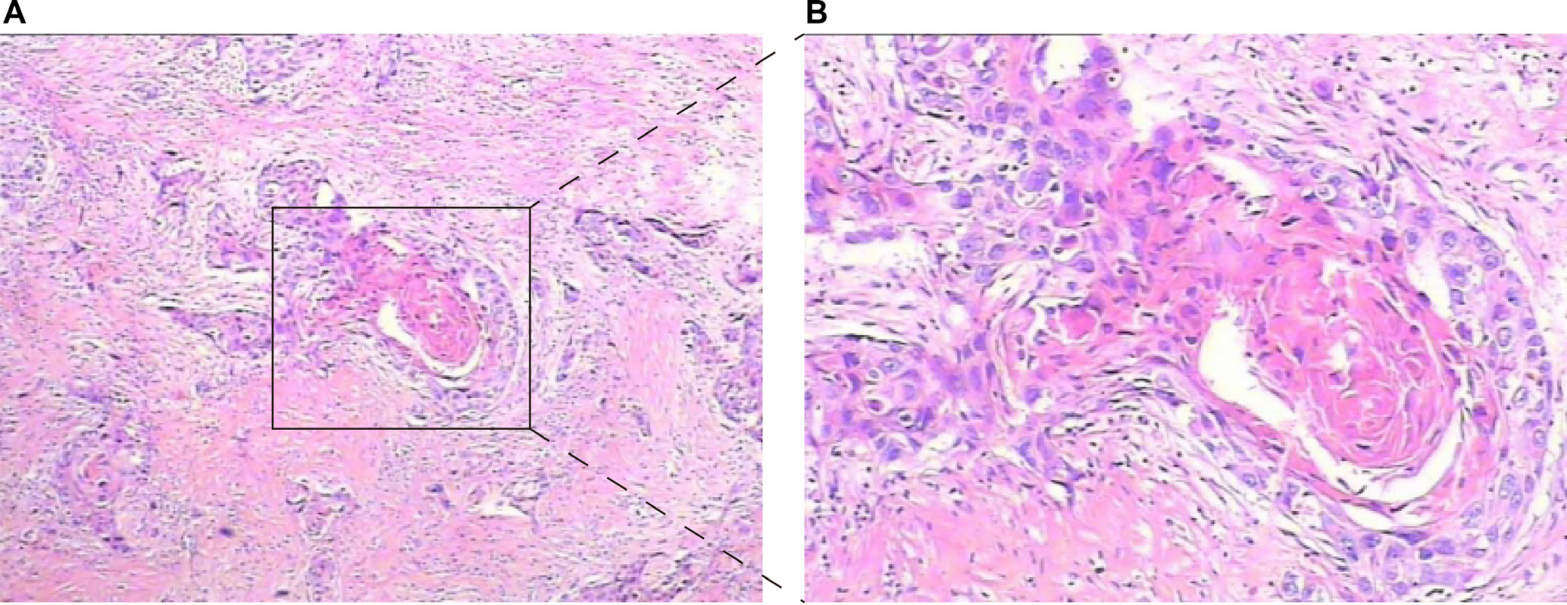
Hematoxylin and eosin staining of bladder urothelial carcinoma with SD ((**A**) Low magnification, (**B**) High magnification).

All surgical specimens were submitted for pathological evaluation. Independent pathologic re-review of three representative slides from each patient was performed by two pathologists on all specimens in order to confirm reported pathologic findings. All specimens were assessed using H&E staining (Figure 1), in which the presence of intercellular bridges or keratinization was indicative of SD. Uncertain pathological diagnosis was confirmed by IHC stains markers against CK20 and CK14.

### Statistical methods

The chi-squared test and Student’s *t*-test were used to evaluate the association between categorical and variables, respectively. Univariate and multivariate logistic regression models were used to analyze. All reported *P* values were two-sided, and a *P* value of ≤ 0.05 was considered to indicate statistical significance. Statistical analysis was performed using SPSS software (Version 18).

## CONCLUSIONS

SD in pT1 bladder urothelial carcinoma is a predictor of poor response for intravesical chemotherapy with epirubicin or hydroxycamptothecine. pT1 group 1 patients should receive early radical cystectomy to prevent disease progression especially for high tumor grade patients or recurrent cases.
